# An Open-Source Test Environment for Effective Development of MARG-Based Algorithms

**DOI:** 10.3390/s21041183

**Published:** 2021-02-08

**Authors:** Ákos Odry

**Affiliations:** Department of Control Engineering and Information Technology, University of Dunaújváros, Táncsics Mihály u. 1, 2400 Dunaújváros, Hungary; odrya@uniduna.hu

**Keywords:** MARG, attitude estimation, complementary filter, inertial measurement unit, Kalman filter, sensor fusion, test environment

## Abstract

This paper presents an open-source environment for development, tuning, and performance evaluation of magnetic, angular rate, and gravity-based (MARG-based) filters, such as pose estimators and classification algorithms. The environment is available in both ROS/Gazebo and MATLAB/Simulink, and it contains a six-degrees of freedom (6 DOF) test bench, which simultaneously moves and rotates an MARG unit in the three-dimensional (3D) space. As the quality of MARG-based estimation becomes crucial in dynamic situations, the proposed test platform intends to simulate different accelerating and vibrating circumstances, along with realistic magnetic perturbation events. Moreover, the simultaneous acquisition of both the real pose states (ground truth) and raw sensor data is supported during these simulated system behaviors. As a result, the test environment executes the desired mixture of static and dynamic system conditions, and the provided database fosters the effective analysis of sensor fusion algorithms. The paper systematically describes the structure of the proposed test platform, from mechanical properties, over mathematical modeling and joint controller synthesis, to implementation results. Additionally, a case study is presented of the tuning of popular attitude estimation algorithms to highlight the advantages of the developed open-source environment.

## 1. Introduction

### 1.1. Relative Localization

Providing accurate pose estimates (i.e., position and attitude results) is a crucial task in the control of agile mobile systems, such as robots. Since the controller and estimator algorithms are linked in closed-loop, therefore, the estimator algorithm should meet important design requirements. Such requirements ensure that the controller algorithm successfully stabilizes the system in the close vicinity of the desired state based on the estimation results [1,2,3]. If these conditions are not satisfied, then the system can easily be driven to unwanted states, which may eventually damage the hardware [4,5,6,7]. As a result, the estimator algorithm should be both analyzed carefully during dynamic conditions and tuned properly in order to provide accurate and robust results [8,9,10,11].

The relative localization problem is solved with microelectromechanical systems-based (MEMS-based) sensors, such as accelerometers, magnetometers, and gyroscopes, in today’s embedded systems. These sensors form the inertial measurement unit (IMU), where a microcontroller processes the sensor data, executes filtering algorithms and functions as an attitude and heading reference system (AHRS) [12]. At the output of the IMU, the attitude is usually provided in Euler angles (roll, pitch, and yaw angles) or quaternion representation of orientation. The filtering algorithm (attitude/pose estimator) is designed such a way to both handle external disturbances effectively and provide properly smoothed signals. There are three types of disturbances that cause radical decrease in attitude estimation quality. On one hand, external accelerations executed during different translational motions prevent the use of the pure gravity vector in attitude calculation; moreover, vibrations occurring during the control of unstable systems also prohibit the obtainment of reliable attitude results [13]. On the other hand, magnetic perturbations are among the key disturbances that make attitude estimation difficult, since ferromagnetic materials alter the local magnetic field in the sensor frame, thereby resulting in inaccurate attitude realizations. Attitude estimator algorithms address the aforementioned issues and execute stochastic-based information fusion or frequency domain processing of MEMS sensor data to provide reliable results. These algorithms are categorized into two main groups, namely Kalman filter (KF) and complementary filter-based (CF-based) approaches, which are prevalent solutions for relative localization problems; usually, both techniques are augmented with deterministic attitude realization methods.

Deterministic approaches use solely gravity and magnetic field observations to solve the Wahba’s problem and provide attitude estimation [14,15]. Among the techniques, the three-axis attitude determination (TRIAD) and QUaternion ESTimator (QUEST) constitute the fundamental approaches; moreover, recent advances offer enhanced solutions, which provide higher reliability [16,17,18]. These approaches work well during static system conditions; however, external disturbances can reduce their performance significantly. Therefore, the high frequency attitude realization of gyroscope signals is incorporated in filter structures to obtain higher robustness in attitude estimation. Gyroscopes are not sensitive to external disturbances, but the numerical integration of angular rate data results in unbounded drift, i.e., only short term accuracy is ensured with angular rate sensors. This short term accuracy is advantageously utilized in both KF and CF structures. CFs use frequency domain information and fuse sensor data by combining the low frequency attitude realization of accelerometer and magnetometer with high frequency attitude results of gyroscope signals via low-pass and high-pass filters, respectively. CFs are characterized by simple structure and easy implementation; therefore, these algorithms are extensively applied in control systems. Among the techniques, the gradient descent algorithm-based (GDA-based) attitude estimator and the explicit complementary filter (ECF) have become popular choices, and their performance is usually considered as benchmark filters in comparative analysis [19,20,21]. Recent approaches in the realm of CFs augment these benchmark filters with adaptive strategies to both obtain higher reliability and handle external disturbances more effectively [22,23,24]. KFs constitute the core algorithms for state estimation of Gaussian stochastic systems. These Bayesian estimators operate on state space models (which describe the system dynamics) and use statistical information to provide state estimates with minimum variance. The magnetic, angular rate, and gravity (MARG) system fits well into stochastic state space models, where the numerically integrated angular rate measurements describe the state propagation, while the accelerometer and magnetometer data are employed in the attitude update (correct) equations. It is common to employ quaternion representation of orientation and describe the system dynamics with a 7-dimensional state space vector, where the temperature dependent gyroscope bias is also involved in the state propagation [25]. Additionally, the update equations are formulated with the application of the rotation matrix (which describes the relationship between the sensor and navigation frames) or the orientation observation is obtained with deterministic approaches [4]. Recent advances extend the basic filter structure with adaptive techniques, in which novel measurement methods, adaptation laws and inference mechanisms, both detect and incorporate the external disturbances into the filter structure, thereby preventing the quality decrease of estimation performance [5,26,27,28]. A recent survey on attitude estimation techniques is provided in Reference [1].

### 1.2. Contribution of the Paper

The performance of each technique discussed in the previous subsection heavily depends on the core parameters that characterize the filter structure. These parameters should be selected carefully in order to both meet the design requirements and provide accurate and robust attitude estimates in the intended application. If the intended application is expected to contain different system conditions (e.g., static states, slow/fast motions and agile movements), then the problem of selecting the proper parameters becomes even more crucial, since the system dynamics influences significantly the MARG-based state estimation process. Usually, it is difficult to derive the appropriate filter parameters (e.g., sensor noise power); moreover, the determination process requires particular equipment and measurement methods. As a result, engineering intuition-based filter tuning is usually performed, which yields only a compromise solution between filter dynamics and accuracy. However, it is also common to use optimization-based techniques to maximize the filter performance [2,25,27,29].

Both approaches discussed above require an environment, which enables the evaluation of filter performances. Namely, the true system states need to be obtained simultaneously with the estimation results in order to determine the estimation quality. The literature offers various solutions for such environments, from camera-based techniques [20,30], over industrial robotic manipulators-based approaches [31,32,33], to experimental apparatuses [2]. These environments enable the researcher to analyze the filter convergence, tune the parameters, observe the issues, and incorporate the observations into the development. Moreover, the optimization of these filter structures can be outlined effectively to obtain maximized filter convergence. These advantages emphasize the need for an universally applicable test environment that enables the execution of the aforementioned tasks.

As a result of the investigation, this paper proposes a novel test environment for effective development of MARG-based algorithms. This test environment includes a six-degrees of freedom (6 DOF) mechanism to execute various external accelerations and vibrations, thereby simulating different system conditions. An MARG unit is attached to the end of the defined kinematic chain of joints; therefore, the environment simultaneously supplies the raw MARG sensor data, along with the true pose (position and orientation) states, during the motion of the system. Moreover, the test environment includes an artificial magnetic perturbation algorithm to generate realistic magnetic disturbance effects during the execution of different test scenarios. This complete environment allows to simulate various real word scenarios and thereby enables both the development and testing of any filter structure. Additionally, the tuning of filter parameters can easily be performed, since the true states of the system are provided in the environment, and even numerical optimization can be employed to obtain maximized filter performance. The author made both the ROS/Gazebo and MATLAB/Simulink implementations publicly available in the Supplementary Online Material [34] in order to help other lab teams in the development of MARG-based algorithms. The proposed test environment has been employed during the development of an attitude estimator algorithm in an earlier work [1]. However, the main components (i.e., the structure of the environment, test bench properties, derivation of models, perturbation algorithms, and applied controllers) which form this comprehensive environment have not been published yet.

The remainder of the paper is organized as follows. Section 2 describes the complete elaboration of the test environment, from system equations, over control synthesis and MARG unit modeling, to implementation results. Section 3 gives a case study on the effective evaluation of attitude filters with the help of the proposed test platform. Finally, Section 4 provides the conclusions and recommendations for future studies.

## 2. Test Environment

To be able to effectively develop, test, and evaluate different relative localization-type algorithms, a test environment is required to be designed. This test environment should both allow to simulate various realistic system behaviors (i.e., static behaviors, dynamic conditions, and external perturbations) and contain realistic sensor models which include manufacturing errors and noise sources. Additionally, the test environment should provide the real system states (ground truth position and orientation), along with the raw, noisy, uncalibrated sensor data, during the execution of different system conditions. These features both enable the evaluation of filter performances and foster the development of novel techniques.

**Figure 1:**
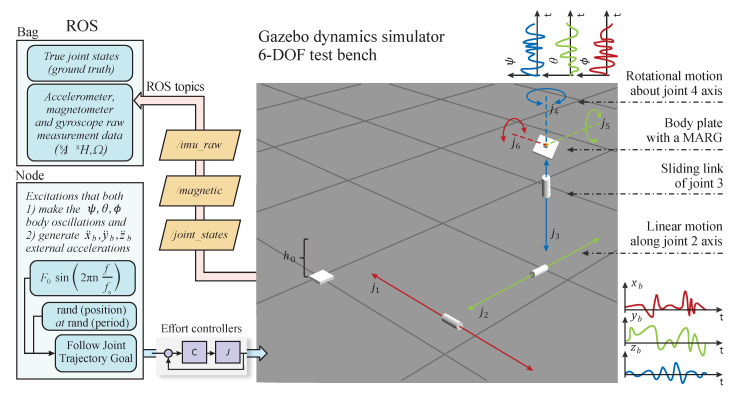
Structure of the test environment in ROS/Gazebo.

A test environment that satisfies the aforementioned requirements is depicted in Figure 1. This environment contains a 6 DOF platform that alters the pose (both position and orientation) of an MARG unit in the 3D space. The closed-loop structure simulates dynamic circumstances, i.e., the 6 DOF platform executes the desired mixture of static and dynamic system behaviors based on the supplied reference signals. Since both the system dynamics and sensor models are included in this environment; therefore, the acquisition of true system states (true position and orientation of the MARG frame), along with raw sensor data, is supported. These measurements contributes to post-processing, such as the analysis of dynamic effect on estimation process, filter performance quantification (i.e., state estimation error determination), and optimization of filter parameters. These attributes form the main advantages of the proposed environment, which has been implemented in both ROS/Gazebo and MATLAB/Simulink. Moreover, video demonstrations of the closed-loop dynamics, along with the ROS/MATLAB-based program packages, have been made publicly available on the author’s website [34].

**Figure 2:**
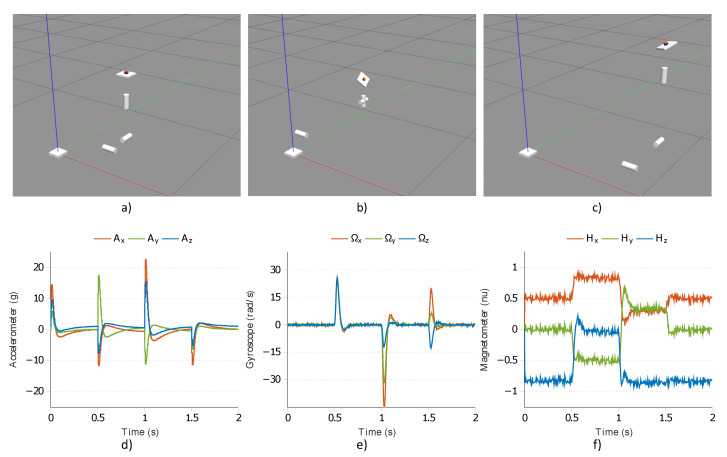
(**a**–**c**) Snapshots during the motion of the 6 six-degrees of freedom (6 DOF) platform; (**d**–**f**) raw measurements of the attached magnetic, angular rate, and gravity (MARG) unit.

The 6 DOF test bench consists of three prismatic joints and three revolute joints. The prismatic joints maintain the desired spatial coordinates of the MARG unit. Moreover, by sliding back and forth, up, and down the sensor frame in the 3D space different accelerating system conditions are simulated via these joints. The revolute joints control the orientation (instantaneous Euler angles) of the sensor frame. These joints can execute both fast and slow rotation motion to simulate vibration and oscillation effects. A plate is attached to the end of this kinematic chain which contains the MARG unit (see the small orange sensor block in Figure 1). Therefore, this 6 DOF platform enables the control and measurement of the pose of sensor frame; moreover, the implemented sensor models provide the instantaneous measurements (raw sensor data) related to the system dynamics simultaneously. This comprehensive framework supplies the necessary environment and data to both generate various system conditions (dynamic spatial motion, along with external accelerations and sensor frame vibrations) and access the raw sensor measurements, along with the true system states, for post-processing and analysis. In the first row of Figure 2, three snapshots of the 6 DOF platform are depicted during the motion in the 3D space; moreover, the generated instantaneous accelerometer, gyroscope, and magnetometer measurements are highlighted in the second row.

### 2.1. Model Equations

Table 1 describes the main parameters of the model. The system dynamics is obtained with the help of the Lagrange-equations [35]:

(1) $$\frac{d}{\;dt}\frac{\partial L}{\partial \dot{q}}-\frac{\partial L}{\partial q}=\tau ,$$

where *q* denotes the vector of generalized coordinates, and L defines the Lagrange function. The Lagrange function is defined as the difference of kinetic and potential energies, i.e., L=K−P. The total kinetic energy of the system is composed of the kinetic energy resulting from translational motions ($T_{t}$, executed by the prismatic joints) and rotational energy ($T_{r}$, generated by the revolute joints) resulting from the oscillation of the body plate, i.e.,

(2) $$\begin{array}{cc} T_{t} & =\frac{1}{2}\left(\left(m_{j,1}+m_{b}\right){\dot{x}}_{b}^{2}+\left(m_{j,2}+m_{b}\right){\dot{y}}_{b}^{2}+\left(m_{j,3}+m_{b}\right){\dot{z}}_{b}^{2}\right),\\ T_{r} & =\frac{1}{2}\left(J_{b,\phi }{\dot{\phi }}^{2}+J_{b,\theta }{\dot{\theta }}^{2}+J_{b,\psi }{\dot{\psi }}^{2}\right). \end{array}$$

By introducing the vector of generalized coordinates as $q={\left(x_{b},y_{b},z_{b},\phi ,\theta ,\psi \right)}^{T}$, the total kinetic energy is formulated in a compact form as:

(3) $$\begin{array}{cc} T & =T_{t}+T_{r}=\frac{1}{2}{\dot{q}}^{T}M_{\mathrm{mass}}\dot{q},\\ M_{\mathrm{mass}} & =\mathrm{diag}\left({\left(m_{j}^{T}+I_{1\times 3}m_{b},J_{b}^{T}\right)}^{T}\right), \end{array}$$

where *I* denotes the identity matrix of size given in subscript, and $m_{j}={\left(m_{j,1},m_{j,2},m_{j,3}\right)}^{T}$, $m_{j,i}$ is the mass of the *i*th link, i={1,2,3}, while $m_{b}$ and $J_{b}={\left(J_{b,\phi },J_{b,\theta },J_{b,\psi }\right)}^{T}$ indicate the mass and moment of inertia of the body plate, respectively. The potential energy stored in the system is:

(4) $$\begin{array}{cc} P= & \left(m_{b}+\sum_{i}^{3}m_{j,i}\right)gh_{0}+\left(m_{b}+m_{j,3}\right)gz_{b}-m_{j,3}gh_{1}, \end{array}$$

where $h_{0}$ is the base height, and $h_{1}$ denotes the distance between the body plate and third prismatic joint (see Figure 1). Based on Equations (3) and (4), the Lagrange function of the system L is derived.

The vector of generalized external forces (τ in Equation (1)) is defined as $\tau ={\left(\tau_{1},\cdots ,\tau_{6}\right)}^{T}$, where $\tau_{i}$ denotes the generalized force acting on the *i*th joint, i=1,…,6. The generalized external force on each joint consists of the external torque $\tau_{a,i}$ produced by the joint driver (motor) and the effect of friction $\tau_{f,i}$, i.e., $\tau_{i}=\tau_{a,i}-\tau_{f,i}$. Each joint is characterized by both static and viscous (damping) frictions; therefore, the friction is modeled with *sliding* and *stuck* state transitions as follows. Initially, the friction state is set to the stuck condition, and state transition occurs at the *i*th joint only if the transmitted motor torque is bigger than the static torque $\tau_{s,i}$, i.e.,

(5) $$\left|\tau_{a,i}\right|>\tau_{s,i}.$$

If the above condition is satisfied, then the sliding mode is activated and viscous friction acts on the joints, i.e.,

(6) $$\tau_{f}=\mathrm{diag}\left(f_{1},\cdots ,f_{6}\right)\dot{q},$$

where $f_{i}$ denotes the viscous friction coefficient for both prismatic and revolute joints (see Table 1). If the *i*th motor torque becomes smaller than the static friction, moreover, the linear velocity (prismatic joints) or angular velocity (revolute joints) is zero, i.e.,

(7) $$\left|\tau_{a,i}\right|\leq \tau_{s,i}\;\wedge \;{\dot{q}}_{i}=0,$$

then the model switches back to stick condition and static friction is activated at the *i*th joint as:

(8) $$\tau_{f,i}=\mathrm{sign}\left(\tau_{a,i}\right)\cdot \min\left(\left|\tau_{a,i}\right|,\tau_{s,i}\right).$$

The evaluation of Equation (1) results in the motion equations of the system in the following form:

(9) $$M\left(q\right)\ddot{q}+V\left(q,\dot{q}\right)=\tau_{a}-\tau_{f},$$

where $M(q)=M_{\mathrm{mass}}$ denotes the 6-by 6 symmetric and positive definite inertia matrix given in Equation (3), while $V(q,\dot{q})={\left(0_{1\times 2},\left(m_{b}+m_{j,3}\right)g,0_{1\times 3}\right)}^{T}$ is the 6-dimensional vector including the potential (gravity) force terms, where 0 denotes the zero vector of the given size. Based on Equation (9), the state-space representation $\dot{x}\left(t\right)=h\left(x,u\right)$ of the plant is obtained. The state vector is $x_{12\times 1}={\left(q,\dot{q}\right)}^{T}$ and the state-space equation is given as:

(10) $$\begin{array}{cc} \dot{x}\left(t\right) & =\left[\begin{array}{c} \dot{q}\\ M{\left(q\right)}^{-1}\left(\tau_{a}-\tau_{f}-V\left(q,\dot{q}\right)\right) \end{array}\right],\\ y(t) & =x(t). \end{array}$$

### 2.2. Control Synthesis

Proportional-integral-derivative (PID) controllers maintain the position regulation of the 6 DOF test bench. The prismatic joints are actuated with force inputs, while torque signals are supplied to the revolute joints. As a result, six force/torque action-type controllers are applied to perform both translational and rotation motion with the test bench in the 3D space. The control requirements are summarized in Table 2. These requirements ensure the execution of extensive dynamic motions, which inherently results in the simulation of external disturbances in the following ranges: ±30g for external accelerations in the 3D space and ±40rad/s range for angular rates to simulate extensive body plate oscillations. The external accelerations are generated by the prismatic joints during the translational motion in the 3D space. Since these joints maintain the position regulation of the MARG unit, therefore, such settling time and overshoot requirements were selected for these joints that generate multiple external acceleration peaks during the position tracking. The body plate oscillations are executed by revolute joints. These joints maintain the angular position of the MARG unit (roll, pitch, and yaw angles). Since the test bench intends to simulate oscillations and vibrations, therefore, both a magnitude smaller settling time is specified and bigger overshoot is allowed for these joints. The control task was to design such position controllers, which satisfy $\lim_{t\to T_{s}}q\left(t\right)=q_{d}$ at given maximum overshoot (Δv), where $q_{d}$ denotes the desired position, and $T_{s}$ is the settling time.

Since the 6 DOF test platform is characterized by fully decoupled dynamics, therefore, each joint controller can be designed separately. The design of these position controllers is based on a third-order reference system ($H_{d}$), which describes the desired dynamics from the input (reference joint coordinate $q_{d}$) to the output (realized joint coordinate *q*) in closed-loop. First, the damping ratio (ξ) and natural frequency ($\omega_{n}$) of a second-order reference system is obtained based on the defined overshoot and settling time values. The dynamics in time domain is given as:

(11) $$\begin{array}{cc} \ddot{q} & +2\xi \omega_{n}\dot{q}+\omega_{n}^{2}q=\omega_{n}^{2}q_{d},\\ \Delta v & =e^{-\frac{\pi \xi }{\sqrt{1-\xi^{2}}}},\;T_{s}\approx \frac{4}{\xi \omega_{n}}. \end{array}$$

Applying the Laplace transformation, the transfer function of the second order system is obtained:

(12) $$H=\frac{\omega_{n}^{2}}{s^{2}+2\xi \omega_{n}s+\omega_{n}^{2}},$$

where *s* is the Laplace variable. This second order system is extended with a first order dynamics, thereby forming the third-order reference system for the *i*th joint in the following form [36,37]:

(13) $$\begin{array}{c} H_{d,i}=\frac{C_{i}G_{p,i}}{1+C_{i}G_{p,i}}=\frac{Q_{i}\left(s\right)}{Q_{d,i}\left(s\right)}=\frac{\omega_{n}^{2}}{\left(1+Ts\right)\left(s^{2}+2\xi \omega_{n}s+\omega_{n}^{2}\right)}, \end{array}$$

where $C_{i}$ and $G_{p,i}$ denote the controller and plant transfer functions, respectively. For the position control problem, PID-type controllers are applied with feed forward-based gravitation acceleration compensation. Namely, for the *i*th joint:

(14) $$\begin{array}{cc} \tau_{a,i}=\; & K_{P,i}\left(q_{d,i}-q_{i}\right)+K_{I,i}\int_{0}^{t}\left(q_{d,i}-q_{i}\right)d\xi -K_{D,i}{\dot{q}}_{i}-V\left(q_{i},{\dot{q}}_{i}\right). \end{array}$$

As a result, the closed-loop dynamics of the *i*th joint is given as:

(15) $$\begin{array}{cc} \; & M_{i}{\ddot{q}}_{i}+f_{i}{\dot{q}}_{i}=K_{P,i}\left(q_{d,i}-q_{i}\right)+K_{I,i}\int_{0}^{t}\left(q_{d,i}-q_{i}\right)d\xi -K_{D,i}{\dot{q}}_{i}, \end{array}$$

where $M_{i}$ denotes the *i*th element of the mass matrix in Equation (3), and $q_{d,i}$ indicates the desired position for the *i*th joint. By differentiating Equation (15), the equivalent third-order system is obtained, and the PID controller parameters can be derived:

(16) $$\begin{array}{cc} q_{d,i} & =\frac{M_{i}}{K_{I,i}}{\dddot{q}}_{i}+\frac{f_{i}+K_{D,i}}{K_{I,i}}{\ddot{q}}_{i}+\frac{K_{P,i}}{K_{I,i}}{\dot{q}}_{i}+q_{i},\\ H_{r,i} & =\frac{Q_{i}\left(s\right)}{Q_{d,i}\left(s\right)}=\frac{K_{I,i}}{M_{i}s^{3}+\left(f_{i}+K_{D,i}\right)s^{2}+K_{P,i}s+K_{I,i}}. \end{array}$$

The derivation of $K_{P,i}$, $K_{I,i}$, and $K_{D,i}$ parameters for the *i*th PID controller is straightforward. Based on the comparison of the reference system $H_{d,i}$ in Equation (13) and realized system $H_{r,i}$ in Equation (16), one obtains:

(17) $$\begin{array}{cc} K_{I,i} & =\frac{\omega_{n}^{2}}{T}M_{i},\\ K_{P,i} & =\left(\omega_{n}^{2}+\frac{2\xi \omega_{n}}{T}\right)M_{i},\\ K_{D,i} & =\left(2\xi \omega_{n}+\frac{1}{T}\right)M_{i}-f_{i}. \end{array}$$

Since the extra time constant in the third-order reference system ($H_{d}$) results in slower closed-loop dynamics than the desired response, therefore, the damping ratio (ξ) and natural frequency ($\omega_{n}$) parameters may require slight tuning after implementation.

**Figure 3:**
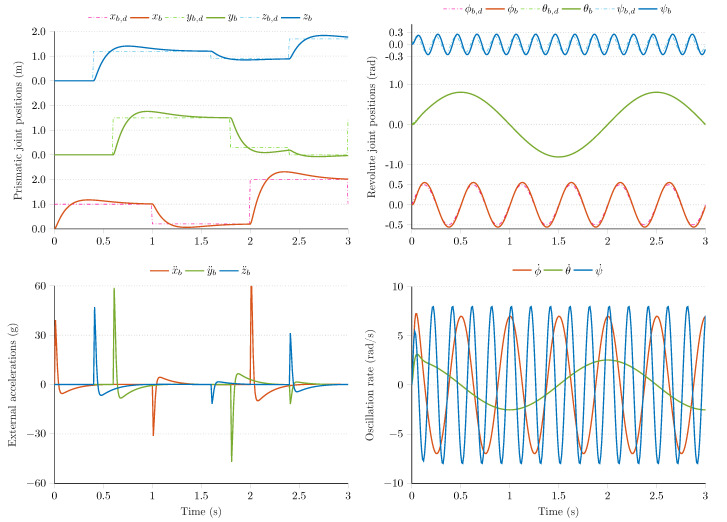
Reference tracking performance of the 6 DOF test bench in closed-loop.

Figure 3 highlights the closed loop dynamics achieved with the derived controllers. In the first row, the reference tracking performance is highlighted for both the prismatic and revolute joints. It can be observed that the prismatic joints maintain the desired $x_{b,d}$, $y_{b,d}$, and $z_{b,d}$ spatial coordinates and fulfill the control requirements. Namely, the settling time for each actuated joint is less than 1 s; moreover, the reference values are tracked with reasonable overshoot (the overshoot is a *must have* requirement, since it results in additional external acceleration, which can be incorporated into the quality verification of estimation algorithms). In the experimental results, the reference values are supplied with approximately 1-s period for the prismatic joints, while the revolute joints are required to track 2Hz, 0.5Hz, and 5Hz sinusoidal signals with different amplitudes to maintain the desired roll, pitch, and yaw angles.

The right side of the first row highlights the performance of actuated revolute joints. Since these joints intend to simulate different oscillation magnitudes; therefore, the joints are characterized by faster dynamics. Based on the figure, it can be concluded that both high and low frequency oscillations are successfully realized by these joints. The reference joint coordinates (desired roll $\phi_{d}$, pitch $\theta_{d}$, and yaw $\psi_{d}$ angles) are tracked with acceptable performance; therefore, the derived PID controllers successfully satisfy the control requirements. In the second row of Figure 3, the generated external accelerations and oscillation rates are highlighted during the motion of the test bench. The external accelerations are in the ±60g range, while the angular rates appear in the ±10rad/s range in this experiment. These values cover an extensive range of dynamic motions, from slow human motion tracking problems to the dynamics of agile mechatronic systems characterized by vibrations, fast maneuvers, and a variety of working conditions. These experimental results prove that the proposed test environment is capable of simulating various system behaviors, which enables the evaluation of different real world scenarios.

**Figure 4:**
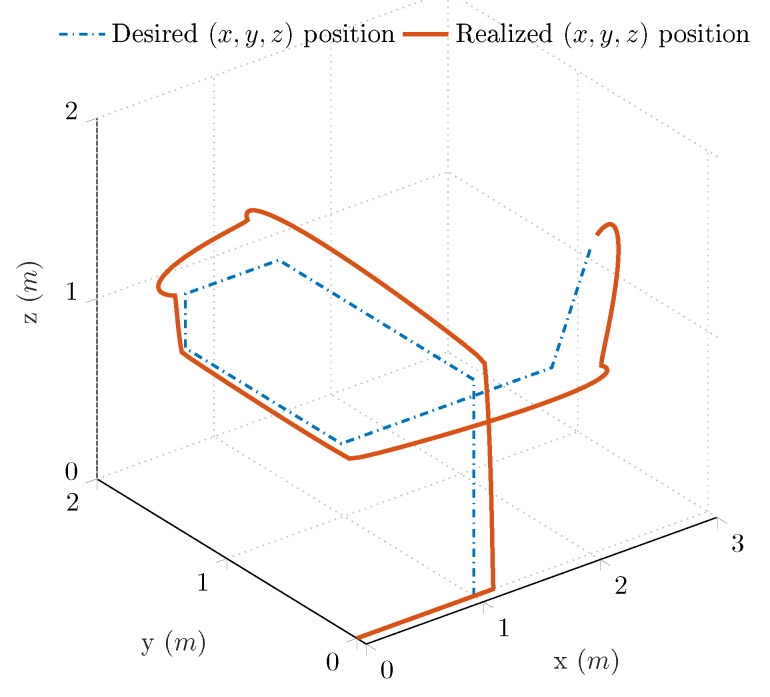
Reference and realized trajectories of the 6 DOF test bench in closed-loop.

Figure 4 depicts the spatial motion of the linear joints during the aforementioned experiment. The blue curve highlights the reference trajectory ($x_{b,d}$, $y_{b,d}$ and $z_{b,d}$), while the red curve shows the realized trajectory during the 3 s-long experiment. An apparent difference can be observed between the blue and red curves, due to the fact that the new desired spatial coordinates have been supplied earlier than the settling of the prismatic joints controllers. Moreover, the desired spatial coordinates are supplied in different time instances; therefore, even if one prismatic joint controller settles, the other two controllers are either in the rising time phase or overshoot state, thereby resulting in discrepancy in either one or two coordinates. However, this discrepancy in trajectory tracking does not influence the task and goal of the environment at any manner, since every control requirement is satisfied; moreover, the test environment enables the generation of different dynamic conditions in the 3D space.

### 2.3. MARG System

The 6 DOF test bench performs various dynamic motions, where the pose of its body plate is altered continuously to simulate both oscillations and external acceleration events. An MARG unit model is attached to the body plate, which provides raw, noisy sensor data during the motion of the system.

The model of MARG unit provides the raw gyroscope $\Omega_{k}$, accelerometer $A_{k}$, and magnetometer $H_{k}$ measurements in each epoch *k* according to the following equations [38]:

(18) $$\begin{array}{cc} \Omega_{k} & =\left(I+\Delta S_{\Omega }\right)M_{\Omega }\omega_{k}+{\bar{\omega }}_{k}+\mu_{k},\\ A_{k} & =\left(I+\Delta S_{A}\right)M_{A}\left(\alpha_{k}+g_{k}\right)+a_{0}+\nu_{k},\\ H_{k} & =\left(I+\Delta S_{H}\right)M_{H}\left(B_{si}h_{k}+b_{hi}\right)+h_{0}+\epsilon_{k}. \end{array}$$

Each sensor model in Equation (18) is characterized by scale factor and misalignment errors. These errors represent the imperfection of manufacturing by the corresponding matrices ΔS and *M*. Moreover, each sensor model provides the measurements as the sum of the real physical quantity, bias term (${\bar{\omega }}_{k}$, $a_{0}$ or $h_{0}$), and Gaussian additive measurement noise ($\mu_{k}$, $\nu_{k}$ or $\epsilon_{k}$). In case of the gyroscope sensor, the real physical quantity is the angular velocity $\omega_{k}$ of the body plate, while the sum of gravitational $g_{k}$ and external acceleration $\alpha_{k}$ constitute the physical quantity of the accelerometer sensor. Additionally, the physical quantity of the magnetometer model is the local magnetic field vector $h_{k}$ disturbed by magnetic soft iron $B_{si}$ and hard iron $b_{hi}$ errors. These magnetometer errors are compensated for in self-calibration procedures [39,40,41].

The aforementioned MARG model enables the generation of realistic raw MEMS sensor data during the motion of the 6 DOF test bench. Therefore, the effects of both external accelerations and sensor frame oscillations are appropriately represented by $\Omega_{k}$, $A_{k}$, and $H_{k}$ sensor outputs.

### 2.4. Magnetic Perturbation Generator

Magnetic fluctuations cannot be generated by Equation (18) in the simulation environment. However, random magnetic perturbations commonly occur in real world scenarios, where the magnetometer output gets disturbed by ferromagnetic objects [42]. Therefore, an essential requirement is to simulate random magnetic fluctuations in the proposed test environment.

An additional algorithm is utilized to generate these artificial magnetic perturbations. The algorithm calculates the output (i.e., the magnetic perturbation) in three steps as follows.

(19) $$\begin{array}{cc} m_{k} & =r_{1},\;k=s_{1},\ldots ,s_{1}+L_{w},\\ m_{k} & =\frac{r_{2}}{L_{w}}\left(\left(k-s_{2}\right)\;\mathrm{mod}\;L_{w}\right),\;k=s_{2},\ldots ,s_{2}+L_{w},\\ m_{k} & =r_{3}\sin\left(2\pi f_{1}\frac{k-s_{3}}{L_{w}}\right),\;k=s_{3},\ldots ,s_{3}+L_{w},\\ m_{k} & =r_{4}\sin\left(2\pi f_{2}\frac{k-s_{4}}{L_{w}}\right),\;k=s_{4},\ldots ,s_{4}+L_{w},\\ m_{k} & =\frac{r_{5}}{\pi }\sin^{-1}\left(\sin\left(\pi \frac{k-s_{5}}{L_{w}}\right)\right),\;k=s_{5},\ldots ,s_{5}+L_{w}, \end{array}$$

(20) $$\begin{array}{cc} M_{l} & =\sum_{k=1}^{L_{m}}m_{k}e^{-j\left(\frac{2\pi l\left(k-1\right)}{L_{m}}\right)},\;l=1,\ldots ,L_{m},\\ h_{l} & =1,\;l=1,\left(\frac{L_{m}}{2}+1\right),\;\mathrm{and}\;h_{l}=2,\;l=2,\ldots ,\frac{L_{m}}{2},\\ m_{a,k} & =m_{r,k}+jm_{i,k}=\frac{1}{L_{m}}\sum_{l=1}^{L_{m}}M_{l}h_{l}e^{j\left(\frac{2\pi k\left(l-1\right)}{L_{m}}\right)},\;k=1,\ldots ,L_{m}. \end{array}$$

**Figure 5:**
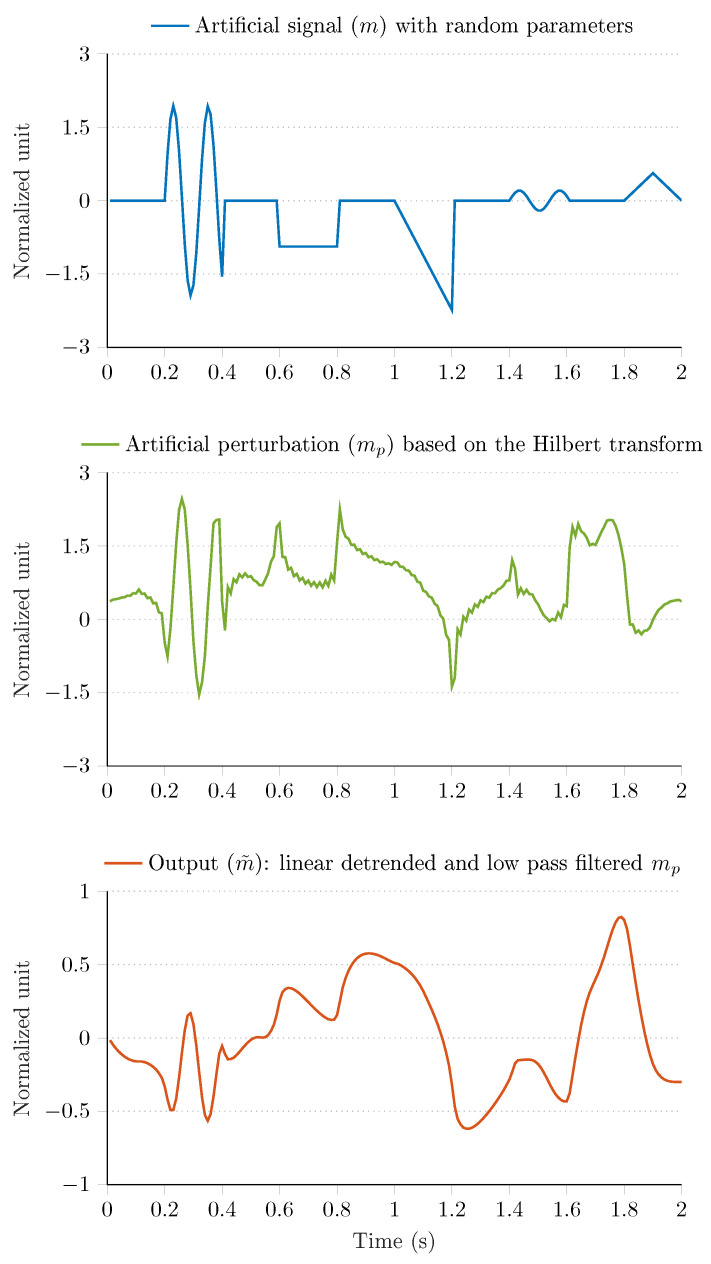
Artificial magnetic perturbation algorithm.

1. Generate an artificial signal by combining five fundamental waveforms. In this algorithm, a square signal, a sawtooth wave, two sinusoidal signals and a triangle wave are combined to generate the artificial signal *m* of length $L_{m}=\Delta tf_{s}$, where Δt denotes the time window, in which magnetic perturbation is to be generated, while $f_{s}$ is the sampling frequency. Namely, initialize $m_{k}=0$ for $k=1,\ldots ,L_{m}$, and then let $L_{w}$ denote the length of each waveform; moreover, a vector $s=\left(s_{1},\ldots ,s_{5}\right)$ containing the start locations of each waveform is generated via a random permutation. As a result, the artificial signal (*m*) is produced with Equation (19), where $r_{i}$ is the random amplitude of the *i*th waveform, i=1,…,5, while $f_{1}$ and $f_{2}$ denote the random frequencies of the sinusoidal signals in $\left[0.5,2.5\right]\;\mathrm{Hz}$ range. The first row of Figure 5 shows the generated artificial signal, where Δt=2 s, $f_{s}=200\;\mathrm{Hz}$ and $L_{w}=L_{m}/10$.
2. Use the artificial signal (*m*) and calculate the analytic signal $m_{a,k}=m_{r,k}+jm_{i,k}$, $k=1,\ldots ,L_{m}$, where $m_{r,k}$ denotes the real part and is the original signal, while $m_{i,k}$ represents the imaginary part, and is the original signal with π/2 phase shift, obtained via Hilbert transformation. In this step, the fast Fourier transform (FFT) M of *m* is calculated, a zero vector *h* of length $L_{m}$ is created and offset (scaling) values are applied, and, finally, the inverse FFT of the element-wise product of *m* and *h* is obtained [43]; see Equation (20). Then, the artificial perturbation $m_{p}$ is generated as the sum of the imaginary part $m_{i}$ and absolute value of the analytic signal $m_{a}$, where the sequence of absolute values is reversed in time (see the outcome of this step in the second row of Figure 5):
  (21) $$m_{p,k}=m_{i,k}+\left|m_{a,l}\right|,$$
3. Extract the continuous linear trend of the artificial perturbation signal by subtracting a straight line ${\hat{m}}_{p}$ from $m_{p}$, where ${\hat{m}}_{p}$ denotes the best linear fit to $m_{p}$ in the least squares sense:
  (22) $$\Delta m_{p}=m_{p}-{\hat{m}}_{p},\;\sum_{k=1}^{L_{m}}{\left(m_{p,k}-{\hat{m}}_{p,k}\right)}^{2}\to \min.$$
  Then, apply low-pass filtration to the detrended signal ($\Delta m_{p}$) via a second order Butterworth infinite impulse response (IIR) filter to obtain the output ($\tilde{m}$):
  (23) $${\tilde{m}}_{k}=\sum_{i=1}^{2}a_{i}{\tilde{m}}_{k-i}+\sum_{j=0}^{2}b_{j}\Delta m_{p,k-j},$$
  where $k=1,\ldots ,L_{m}$; moreover, $a_{i}$ and $b_{i}$ filter coefficients are obtained based on the cutoff frequency of the filter. In this algorithm, the cutoff frequency was selected to be 5Hz. The output of the algorithm is shown in the third row of Figure 5.

**Figure 6:**
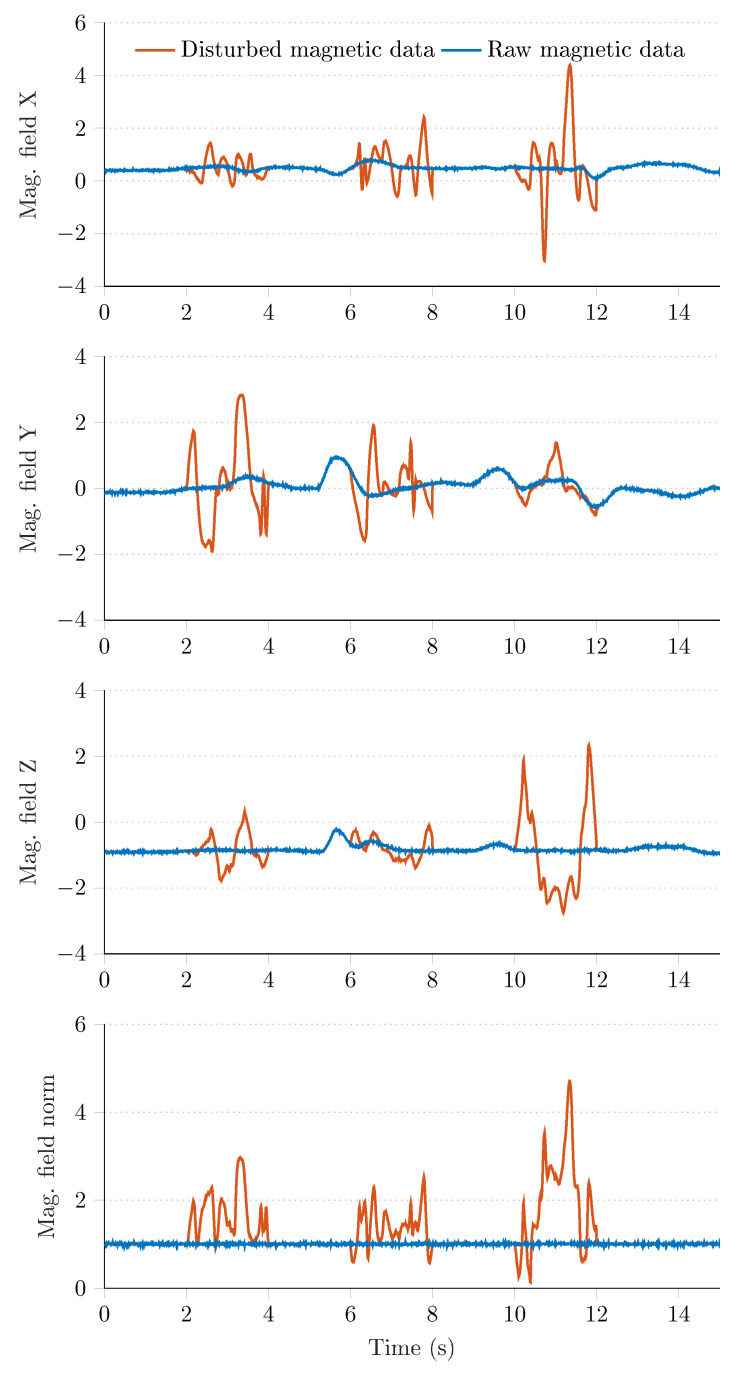
Magnetometer data before (blue curves) and after (red curves) the artificial magnetic perturbations.

Figure 5 highlights that the aforementioned algorithm produces realistic magnetic perturbation signals; therefore, it can be effectively used in the test environment to generate various real world scenarios, where the magnetometer data is disturbed due to environmental changes, such as the effect of ferromagnetic materials. Figure 6 illustrates the application of the proposed algorithm on raw magnetometer data. The blue curves indicate the raw undisturbed magnetometer measurements, while the red curves indicate particular sections, where the measurement was disturbed by the proposed algorithm. It can be seen that the algorithm altered the raw sensor signals three times during the 15 s-long measurement. In the last row of Figure 6 the magnitude of magnetometer data is shown, where the red curve highlights the effect of the perturbation algorithm (i.e., the norm of measurements has increased to approximately five times of the raw data norm during the perturbation events). These figures demonstrate that the algorithm provides useful output for simulating random realistic magnetic perturbations.

### 2.5. Implementation Results

The proposed 6-DOF test bench, along with its closed-loop architecture, MARG unit model, and magnetic perturbation algorithm, as a complete simulation environment, has been implemented in both MATLAB/Simulink and ROS/Gazebo. These implementations have been made publicly available in the Supplementary Online Material [34] with the aim to share the test environment with other researchers who’s work is focused on MEMS MARG unit-based algorithms. Appendix A and Appendix B describe the complete procedure of the usage of the proposed test environment.

**Figure 7:**
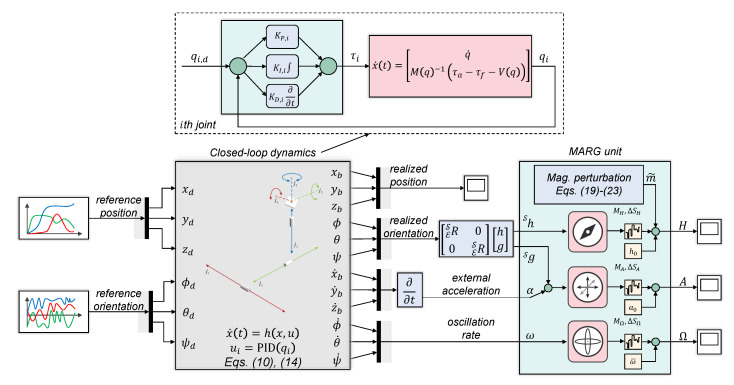
The test environment in MATLAB/Simulink.

Regarding the MATLAB-based implementation, the state-space dynamics of the 6 DOF test platform (Equation (10)) has been implemented with a Simulink S-function block. Each joint is controller in closed-loop; therefore, six position input effort output-type controllers have been implemented (Equation (14)) to maintain the desired joint coordinates. The PID parameters have been obtained using Equation (17). The MARG unit is connected to the end of the kinematic chain and is composed of accelerometer and gyroscope Simulink blocks, along with a custom-made magnetometer model (see Equation (18)). In the current implementation, it is assumed that the sensor models are calibrated (bias terms are zero vectors); moreover, zero scaling and no misalignment are considered in Equation (18). However, these values can easily be modified in the corresponding Simulink sensor blocks, if more advanced problems need to be examined. Figure 7 shows the implemented model in MATLAB/Simulink.

In ROS/Gazebo environment, the dynamics of the 6 DOF test bench is implemented in a Universal Robotic Description Format (URDF) file [44]. This XML file describes all elements of the test environment, from kinematic and dynamic properties (mass, inertia, friction parameters, and joint ranges), over collisions to frame positions and velocities. The derived PID controllers have been implemented and loaded via the *controller manager* ROS package. Effort action-based controllers are defined to drive the joints in closed-loop. The *ros controllers* meta-package provides the environment to implement these low level controllers. As it is given in Equation (14), these controllers are driven with desired position inputs and supply torque outputs to the joints. The state vector $x={\left(q,\dot{q}\right)}^{T}$ is obtained by the *joint state controller*, which is a sensor controller and supplies the true states of the system continuously (true joint positions, velocities, and joint efforts without measurement noise). The MARG unit is connected to the body plate of the test platform and is implemented with Gazebo sensor plugins [45]. The positions, frames, and noise properties of these sensors are defined in the aforementioned URDF file. Figure 1 shows the implemented model in ROS/Gazebo environment.

## 3. Case Study: Evaluation and Tuning of Attitude Filters

This section intends to show that the proposed environment fosters the effective analysis of MARG-based algorithms. Namely, the test platform enables (i) to observe the impact of different dynamic conditions on the estimation performance, (ii) the tuning of filter parameters, (iii) to detect robustness issues, and, finally, (iv) the complete development and evaluation MARG-based algorithms.

The case study demonstrates the performance evaluation and tuning of attitude filters in the following subsections. As it was discussed in the introduction, the task of attitude estimators is to provide both accurate and robust estimation results even if external disturbances (external acceleration, vibration, and magnetic perturbation) occur during the motion of the dynamical system. Therefore, the performance of these filters can be tested in such environment where the aforementioned disturbances commonly appear with different magnitudes and frequencies. The proposed test environment is completely suitable for the simulation of these system conditions.

### 3.1. Algorithms

Three popular attitude filters are discussed in the analysis, namely the ECF [19], GDA [20], and standard extended Kalman filter (EKF) [46] algorithms are evaluated with the help of the test environment. The implementations of these algorithms are widely used and available in both ROS/Gazebo [22,47,48] and MATLAB/Simulink environments [49,50]. Since the algorithms have been described in detail in earlier works [1,2,4,5,32,51,52], therefore, only the key steps of the algorithms are introduced as follows.

#### 3.1.1. Explicit Complementary Filter

The algorithm in Reference [19] derives a quaternion-based CF, in which the error between the measured and estimated directions of gravity and magnetic field vectors is calculated. Then, a PI controller is used to correct the gyroscope signal, which finally drives the quaternion propagation:

(24) $$\begin{array}{cc} e_{k} & ={}^{S}A_{k}\times {}^{S}\hat{A}\left({\hat{q}}_{k}\right)+{}^{S}H_{k}\times {}^{S}\hat{H}\left({\hat{q}}_{k}\right),\\ {\hat{\Omega }}_{k} & =\Omega_{k}+K_{p}e_{k}+K_{i}\sum_{i=1}^{k}e_{i}T_{s},\\ {\hat{q}}_{k+1} & ={\hat{q}}_{k}+\frac{T_{s}}{2}Q\left({\hat{q}}_{k}\right)\left[\begin{array}{c} 0\\ {\hat{\Omega }}_{k} \end{array}\right], \end{array}$$

where $T_{s}$ is the sampling period, ${}^{S}A_{k}$ and ${}^{S}H_{k}$ are the measurements in the sensor frame, and ${}^{S}{\hat{A}}_{k}={}_{E}^{S}{\hat{q}}_{k}⊗{}^{E}g⊗{}_{E}^{S}{{\hat{q}}_{k}}^{*}$ and ${}^{S}{\hat{H}}_{k}={}_{E}^{S}{\hat{q}}_{k}⊗{}^{E}h⊗{{}_{E}^{S}{\hat{q}}_{k}}^{*}$ denote the estimated vectors, while $Q\left({\hat{q}}_{k}\right)$ indicates the quaternion matrix [53]. The derivation of these quaternion-based expressions have been described in detail in an earlier work [1]. The filter is characterized by two parameters, these are the PI controller gains $K_{p}$ and $K_{i}$.

#### 3.1.2. Gradient Descent-Based Attitude Filter

The algorithm in Reference [20] also uses quaternion representation of orientation. The accelerometer and magnetometer measurements are employed in a gradient descent algorithm to obtain the quaternion derivative related to the gyroscope measurement error. As a result, a drift corrective step is executed to maintain the gyroscope-based quaternion propagation:

(25) $$\begin{array}{cc} f_{k} & =\left[\begin{array}{c} {}^{S}\hat{A}\left({\hat{q}}_{k}\right)-{}^{S}A_{k}\\ {}^{S}\hat{H}\left({\hat{q}}_{k}\right)-{}^{S}H_{k} \end{array}\right],\\ ▿f_{k} & =J_{k}^{T}f_{k},\\ {\hat{q}}_{k+1} & ={\hat{q}}_{k}+T_{s}\left(\frac{1}{2}Q\left({\hat{q}}_{k}\right)\left[\begin{array}{c} 0\\ \Omega_{k} \end{array}\right]-\beta \frac{▿f_{k}}{∥f_{k}∥}\right), \end{array}$$

where $J_{k}$ is the Jacobian matrix of the objective function $f_{k}$, and β denotes the learning rate. This algorithm is a single-gain (β) attitude filter.

#### 3.1.3. Attitude Estimation with Extended Kalman Filter (EKF)

The EKF employs the gyroscope-based quaternion propagation to obtain the a priori state estimate ${\hat{x}}_{k}^{-}={\left({\hat{q}}_{k}^{-},{\hat{\bar{\omega }}}_{k}^{-}\right)}^{T}$ in the so-called predict phase, and then these estimation results are updated with the accelerometer and magnetometer readings $z_{k}={\left({}^{S}A_{k},{}^{S}H_{k}\right)}^{T}$ in the measurement update phase of the algorithm. The state propagation includes both the quaternion ($q_{k}$) and random walk process of gyro-bias (${\bar{\omega }}_{k}$), while the update equations are based on the relationship between the reference vectors and sensor measurements:

(26) $$\begin{array}{cc} {\hat{q}}_{k}^{-} & ={\hat{q}}_{k-1}+\frac{T_{s}}{2}Q\left({\hat{q}}_{k-1}\right)\left[\begin{array}{c} 0\\ \Omega_{k}-{\hat{\bar{\omega }}}_{k-1} \end{array}\right],\\ P_{k}^{-} & =\Phi P_{k-1}\Phi^{T}+Q,\\ G_{k} & =P_{k}^{-}H^{T}{\left(HP_{k}^{-}H^{T}+R\right)}^{-1},\\ {\hat{z}}_{k}^{-} & ={\left({}^{S}\hat{A}\left({\hat{q}}_{k}^{-}\right),{}^{S}\hat{H}\left({\hat{q}}_{k}^{-}\right)\right)}^{T},\\ {\hat{x}}_{k} & ={\hat{x}}_{k}^{-}+G_{k}\left(z_{k}-{\hat{z}}_{k}^{-}\right),\\ P_{k} & =\left(I-G_{k}H\right)P_{k}^{-}. \end{array}$$

In Equation (26), $\hat{x}$ is the state estimate, and Φ and *H* denote the Jacobians of state dynamics and measurement update equations, respectively, while *P* is the error covariance and *G* is the Kalman gain. Moreover, *Q* and *R* denote the state and measurement noise covariance matrices, which mainly determine the performance of the EKF.

### 3.2. Experimental Results

In the conducted experiments, the 6 DOF test platform executed various system behaviors in order to evaluate the filter convergences in different scenarios. External accelerations and sensor frame oscillations have been generated by supplying random desired spatial coordinates to the PID controllers of the three prismatic joints. Simultaneously, diverse sinusoidal signals as reference joint coordinates were supplied to the PID controllers of the three revolute joints to maintain a variety of sensor frame oscillations, where both the amplitude and frequency coefficients were randomly generated. As a result, the closed-loop system of the 6 DOF mechanism executed an extensive range of dynamic motions in the 3D space. Moreover, the proposed magnetic perturbation algorithm generated random pose-based disturbance events, as well. During these system motions, the test environment recorded the true system states (ground truth position and orientation of the sensor frame), along with the raw, noisy MARG sensor data. Therefore, the collected database enabled to both evaluate and tune the attitude estimator algorithms.

**Figure 8:**
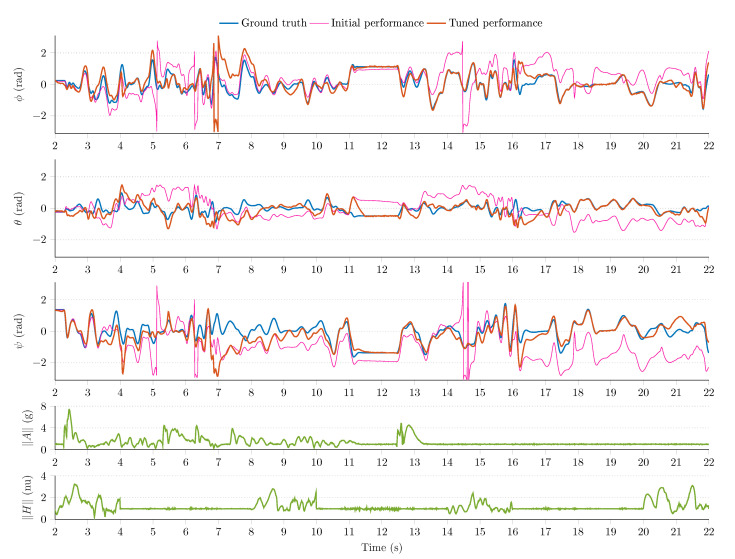
Initial and tuned performance of the gradient descent-based attitude filter.

**Figure 9:**
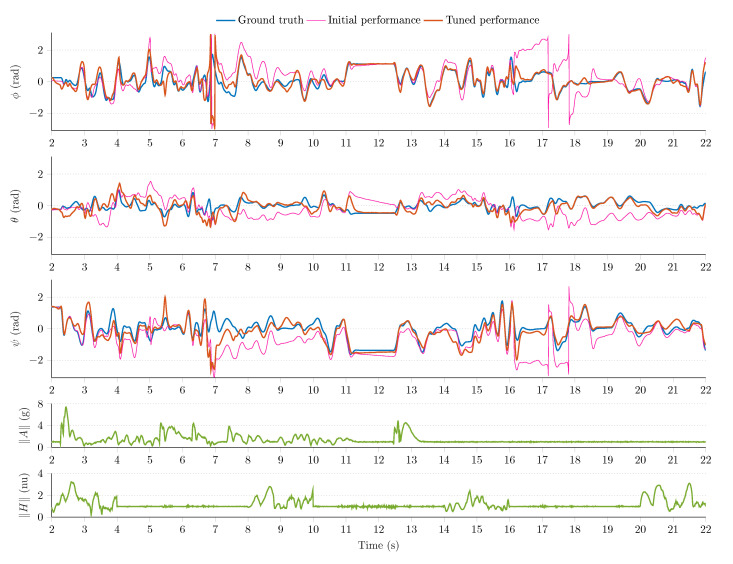
Initial and tuned performance of the explicit complementary filter.

**Figure 10:**
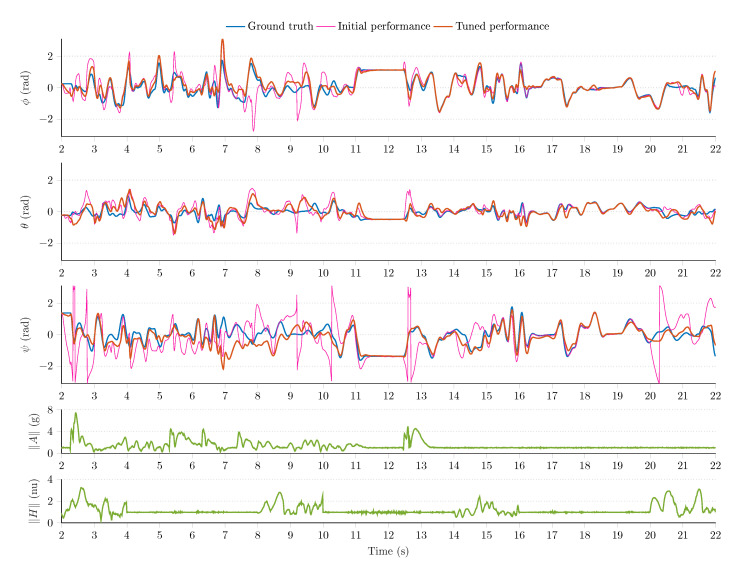
Initial and tuned performance of the standard extended Kalman filter (EKF).

Figure 8, Figure 9 and Figure 10 show a sample measurement set obtained in the simulation environment. The sampling frequency was set to $f_{s}=200\;\mathrm{Hz}$; in the MARG model (see Equation (18)), the standard deviations were defined as follows: $\mu =0.0224\;\left(\mathrm{rad}/s\right)/\sqrt{\mathrm{Hz}}$ for the gyroscope model, $\nu =0.0224\;\left(m/s^{2}\right)/\sqrt{\mathrm{Hz}}$ for the accelerometer output, and $\epsilon =0.0022\;\mathrm{nu}/\sqrt{\mathrm{Hz}}$ for the magnetometer readings (normalized unit, nu). The magnetic perturbation algorithm was executed for random Δt=2 s time windows. The 6 DOF platform performed both static and dynamic motions in the following ranges: oscillations were generated with revolute joints in the ±50rad/s angular velocity range, ±4g external accelerations were executed by the prismatic joints, and magnetic perturbations were applied in the 0–4 nu disturbance range.

The MARG sensor data (the output of the simulation environment) drove the attitude filter algorithms (Equations (24)–(26). The outputs of attitude filters were observed; moreover, the tuning of each filter was performed based on the heuristic techniques developed in References [1,2,54]. Figure 8 shows the GDA output, and Figure 9 highlights the performance of the ECF algorithm, while Figure 10 illustrates the output of the EKF algorithm. The first three rows of each figure highlight the ground truth Euler angles (true roll ϕ, pitch θ, and yaw ψ angles are indicated with blue curves); moreover, the initial (pink curves) and tuned (red curves) filter performances are shown in these subplots. The fourth row of each figure depicts the magnitude of accelerometer readings; therefore, both the static and dynamic system behaviors can be identified. Additionally, the norm of magnetometer data is depicted in the fifth row, which enables the identification of magnetic disturbance events.

It can be observed that the test environment effectively fostered the tuning procedure of each filter algorithm. The initial filter outputs (pink curves) contained notable estimation errors; therefore, the default filter parameters did not contribute to an acceptable filter performance during the executed dynamic motions. However, the tuning of filter parameters could easily be performed in the environment, which resulted in improved filter performances. The effects of external accelerations, vibrations, and magnetic perturbations can also be observed in the measurement results. These external disturbances significantly decrease the performance of each filter during the first 12 s in the experiment. These effects can be handled more effectively in adaptive filter structures. The analysis of adaptive filters exceeds the scope of this article, but, in fact, the test environment aims to effectively support the development and evaluation of such novel techniques.

The test environment also enables the evaluation of filter convergence in terms of mean squared error (MSE) and standard deviation (STD) of attitude estimation errors ($e_{\phi ,k}=\phi_{k}-{\hat{\phi }}_{k}$, $e_{\theta ,k}=\theta_{k}-{\hat{\theta }}_{k}$, and $e_{\psi ,k}=\psi_{k}-{\hat{\psi }}_{k}$, where ${\hat{\phi }}_{k}$, ${\hat{\theta }}_{k}$, and ${\hat{\phi }}_{k}$ denote the outputs, estimated Euler angles, of the implemented filter algorithms in epoch *k*). Moreover, a cumulated performance index can be defined with Equation (27) to quantify the overall performance (*N* is the length of the measurement). The evaluation of these performance indexes is summarized for each filter in Table 3. Based on the results, it can be observed that each filter provides significantly enhanced performance with the tuned parameters.

(27) $$\begin{array}{c} F=\sqrt[{3}]{\left(\frac{\sum_{k=1}^{N}e_{\phi ,k}^{2}}{N}\right)\left(\frac{\sum_{k=1}^{N}e_{\theta ,k}^{2}}{N}\right)\left(\frac{\sum_{k=1}^{N}e_{\psi ,k}^{2}}{N}\right)} \end{array}.$$

Among the algorithms, the GDA was characterized by the largest performance index (F=0.1812) in the experiment (the smaller the value, the better the performance). This outcome can be related to the single (constant) gain property of the algorithm. The dynamic circumstances made the GDA provide large estimation errors around 7 s in Figure 8, which resulted in the biggest performance index in the analysis. The ECF showed a more robust filter performance with F=0.1471 performance index. The tuned PI controller enabled the filter to overcome the impact of external disturbances more effectively; however, notable estimation errors can be observed during the dynamic behavior of the system. The EKF algorithm yielded the most robust filter performance during the experiment (F=0.1033). This algorithm was characterized by four parameters (i.e., noise statistics related to quaternion propagation, bias walk process, accelerometer observations, and magnetometer measurements); therefore, its flexibility allowed to both effectively suppress the effects of disturbances and provide superior filter performance. However, the performance of EKF algorithm also decreased during the dynamic motions, as can be observed during the first 10-s period. The effective handling of these disturbances is performed by adaptive sensor fusion structures.

This subsection demonstrated the use of the proposed test environment for the analysis and tuning of common attitude filters. The test environment can be universally applied for any type of MARG-based algorithm, where external accelerations, vibrations, and magnetic perturbations commonly occur, and their effect should be carefully addressed (and incorporated) in the development of novel algorithms.

## 4. Conclusions

This paper presented an universally applicable test environment for development, testing, and performance evaluation of MARG-based filtering techniques, such as pose estimators and classification algorithms. The test environment contains a closed-loop controlled 6 DOF dynamical system to alter the pose of an MARG unit in the 3D space and thereby simulate various system behaviors. Effort action-based joint controllers are employed to execute realistic external accelerations and vibrations by the 6 DOF platform; moreover, an artificial magnetic perturbation algorithm is applied to generate realistic magnetic disturbances. The simulation environment executes the desired dynamical motions and simultaneously supplies the true system states (ground truth values), along with the raw sensor measurements. As a result, the proposed environment provides effective help in the development of filtering techniques, since a big variety of system conditions can be both simulated and evaluated. The environment has been implemented in both ROS/Gazebo and MATLAB/Simulink and is publicly available online. Future work will include the analysis of advanced conditions, where the impact of static friction, sensors frame misalignment, and scaling errors will also be evaluated during the state estimation procedures.

## Figures and Tables

**Table 1:** Notation of basic test bench parameters.

Parameter	Symbol (Unit)	Value
Mass of link 1 and 2	$m_{j,1}$, $m_{j,2}$ (kg)	500
Mass of link 3	$m_{j,3}$ (kg)	200
Mass of the body plate	$m_{b}$ (g)	200
Inertia of the body plate (ϕ, θ)	$J_{b,\phi }$, $J_{b,\theta }$ (kg${\mathrm{mm}}^{2}$)	60
Inertia of the body plate (ψ)	$J_{b,\psi }$ (kg${\mathrm{mm}}^{2}$)	40
Viscous friction coefficient (joint 1–3)	$f_{1}$, $f_{2}$, $f_{3}$ (Ns/m)	100
Viscous friction coefficient (joint 4–6)	$f_{4}$, $f_{5}$, $f_{6}$ (Nms/rad)	0.001
Static friction (joint 1–3)	$\tau_{s,1}$, $\tau_{s,2}$, $\tau_{s,3}$ (N)	0
Static friction (joint 4–6)	$\tau_{s,4}$, $\tau_{s,5}$, $\tau_{s,6}$ (Nm)	0

**Table 2:** Control requirements related to the desired system dynamics.

Parameter	Symbol (Unit)	Value
External acceleration executed by joint 1–3	$\alpha_{ext,1,2,3}$ (g)	30
Oscillation rate executed by joint 4–6	$\Omega_{4,5,6}$ (rad/s)	40
Overshoot for joint 1–3	$\Delta v_{1,2,3}$ (%)	20
Settling time for joint 1–3	$T_{s}$ (s)	1
Overshoot for joint 4–6	$\Delta v_{4,5,6}$ (%)	40
Settling time for joint 4–6	$T_{s}$ (s)	0.3

**Table 3:** Outcome of filter analysis. The bold values indicate the tuned filter performances.

	ECF		GDA		EKF	
	Initial	Tuned	Initial	Tuned	Initial	Tuned
F	0.5237	**0.1471**	1.1950	**0.1812**	0.2891	**0.1033**
MSE (ϕ)	0.4405	0.1831	1.2361	0.2349	0.2351	0.0856
STD (ϕ)	0.6307	0.4275	0.9103	0.4835	0.4848	0.2881
MSE (θ)	0.3713	0.0773	0.5610	0.0977	0.1068	0.0676
STD (θ)	0.5996	0.2756	0.7410	0.3069	0.3184	0.2593
MSE (ψ)	0.8784	0.2248	2.4607	0.2589	0.9618	0.1906
STD (ψ)	0.6384	0.4476	1.0554	0.4871	0.9774	0.4115

## Data Availability

Not applicable.

## Supplementary Materials

The complete project is available online at http://appl-dsp.com/faekf.

## Appendix A. Usage of the package in MATLAB/Simulink

After downloading the package from Reference [34], the *start* MATLAB file should be executed in the *simulink* folder. This script loads the system parameters (e.g., inertia and mass properties of the joints, friction coefficients, and PID controller parameters) and opens the Simulink simulation environment (see Figure 7). The researcher can freely change the length of simulation (*tsim*), sampling time (*Tsampling*), and time window for magnetic perturbation (*dt*) parameters to evaluate different dynamic scenarios, as well. Then, the desired position and orientation values should be supplied to the 6 DOF model (see the left side of Figure 7); these reference values will be realized by the dynamic system with the help of the closed-loop driven linear and revolute joints. The desired values can be supplied by different Simulink blocks, i.e., from simple *Repeating Sequence Generator* block, over *Signal Generator* block, to custom made signal waves (*From Workspace block*) can be utilized for the generation of reference values. The properties of MARG system can also be modified by editing the parameters of *Gyroscope*, *Accelerometer*, and *Magnetometer* blocks (see the right side of Figure 7). Namely, the natural frequency and damping ratio parameters, scaling, and misalignment errors, as well as the noise power for each axis (see Equation (18)), can be freely modified for each sensor block to observe different system scenarios. Once the parameters and input reference values are set up, the simulation environment can be executed. The outputs of the simulation are the true system states (position in *posXYZ*, orientation in *angleXYZ*, angular velocity in *oscRate*, linear acceleration in *extAccel*, and angular acceleration in *extAngularAccel*) and the realistic MARG sensor data (accelerometer in *aMeas*, gyroscope in *gMeas*, and magnetometer in *mMeas*). These outputs can be used to both effectively evaluate filter algorithms and tune filter parameters.

## Appendix B. Usage of the package in ROS/Gazebo

The package [34] should be cloned and built in the *catkin workspace*. The 6 DOF test environment node is started with the *spawn testenv* launch file. This launch file uses the URDF file to construct both the kinematic chain and dynamics of the 6 DOF system (see the description in Section 2.5). The researcher can freely change the parameters of the test environment (i.e., from dimension of links, over mass parameters and joint limits, to MARG errors, noise powers, and sampling time) in the *testenv xacro* file in the *urdf* folder. Therefore, the aforementioned launch file spawns the dynamic system in Gazebo with the selected parameters; moreover, it adds a PID controller to each joint with the help of the *controller manager* package. The PID controller parameters can also be altered in the *testenv trajectory control yaml* file in the *config* folder. Once the spawning of the test environment is finished, the Gazebo simulator utilizes its physics engines to continuously update the instantaneous system states via ROS topics. The researcher can observe and monitor these instantaneous values in the *rqt* graphical user interface by subscribing to the *joint states*, *imu raw*, and *magnetic* ROS topics (see both Figure 1 and video demonstrations in Reference [34]). Moreover, the researcher can generate the database of both real system states and raw MARG data with the help of the *rosbag* tool. Finally, the *exec traj* node is utilized to supply the desired position and orientation values to the closed-loop architecture of the 6 DOF system. This node creates a ROS client to the *follow joint trajectory* topic for moving simultaneously the linear and revolute joints according to the selected reference values. In this node, the researcher can freely select the way points for each joint to be realized, thereby defining the custom spatial movements to be executed by the dynamic system. As a result, the PID controllers are supplied by the reference values, and the Gazebo simulator simulates the intended system behavior. The instantaneous measurements are collected in the database during the spatial movements by the *rosbag* tool; therefore, both the filter evaluation and parameter tuning can be effectively realized.

## References

[1] Odry Á., Kecskes I., Sarcevic P., Vizvari Z., Toth A., Odry P. (2020). A Novel Fuzzy-Adaptive Extended Kalman Filter for Real-Time Attitude Estimation of Mobile Robots. Sensors.

[2] Odry Á., Fullér R., Rudas I.J., Odry P. (2018). Kalman filter for mobile-robot attitude estimation: Novel optimized and adaptive solutions. Mech. Syst. Signal Process..

[3] Colorado J., Perez M., Mondragon I., Mendez D., Parra C., Devia C., Martinez-Moritz J., Neira L. (2017). An integrated aerial system for landmine detection: SDR-based Ground Penetrating Radar onboard an autonomous drone. Adv. Robot..

[4] Zhang T., Liao Y. (2017). Attitude measure system based on extended Kalman filter for multi-rotors. Comput. Electron. Agric..

[5] Roh M.S., Kang B.S. (2018). Dynamic Accuracy Improvement of a MEMS AHRS for Small UAVs. Int. J. Precis. Eng. Manuf..

[6] Odry Á., Fullér R., Rudas I.J., Odry P. (2020). Fuzzy control of self-balancing robots: A control laboratory project. Comput. Appl. Eng. Educ..

[7] Martínez-Prado M.A., Rodríguez-Reséndiz J., Gómez-Loenzo R.A., Herrera-Ruiz G., Franco-Gasca L.A. (2018). An FPGA-based open architecture industrial robot controller. IEEE Access.

[8] Hashim H.A., Eltoukhy A.E.E. (2021). Landmark and IMU Data Fusion: Systematic Convergence Geometric Nonlinear Observer for SLAM and Velocity Bias. IEEE Trans. Intell. Transp. Syst..

[9] Wen X., Liu C., Huang Z., Su S., Guo X., Zuo Z., Qu H. (2019). A First-Order Differential Data Processing Method for Accuracy Improvement of Complementary Filtering in Micro-UAV Attitude Estimation. Sensors.

[10] Liu S., Lyu P., Lai J., Yuan C., Wang B. (2019). A fault-tolerant attitude estimation method for quadrotors based on analytical redundancy. Aerosp. Sci. Technol..

[11] Khankalantary S., Rafatnia S., Mohammadkhani H. (2019). An adaptive constrained type-2 fuzzy Hammerstein neural network data fusion scheme for low-cost SINS/GNSS navigation system. Appl. Soft Comput..

[12] Lee J.K., Park E.J., Robinovitch S.N. (2012). Estimation of attitude and external acceleration using inertial sensor measurement during various dynamic conditions. IEEE Trans. Instrum. Meas..

[13] Cruz-Miguel E.E., García-Martínez J.R., Rodríguez-Reséndiz J., Carrillo-Serrano R.V. (2020). A New Methodology for a Retrofitted Self-tuned Controller with Open-Source FPGA. Sensors.

[14] Wu J., Shan S. (2019). Dot Product Equality Constrained Attitude Determination from Two Vector Observations: Theory and Astronautical Applications. Aerospace.

[15] Markley F.L., Crassidis J.L. (2014). Fundamentals of Spacecraft Attitude Determination and Control.

[16] Liu F., Li J., Wang H., Liu C. (2014). An improved quaternion Gauss–Newton algorithm for attitude determination using magnetometer and accelerometer. Chin. J. Aeronaut..

[17] Fourati H., Manamanni N., Afilal L., Handrich Y. (2010). A nonlinear filtering approach for the attitude and dynamic body acceleration estimation based on inertial and magnetic sensors: Bio-logging application. IEEE Sens. J..

[18] Wu J., Zhou Z., Fourati H., Cheng Y. (2018). A super fast attitude determination algorithm for consumer-level accelerometer and magnetometer. IEEE Trans. Consum. Electron..

[19] Mahony R., Hamel T., Pflimlin J.M. (2008). Nonlinear complementary filters on the special orthogonal group. IEEE Trans. Autom. Control.

[20] Madgwick S.O., Harrison A.J., Vaidyanathan R. Estimation of IMU and MARG orientation using a gradient descent algorithm. Proceedings of the 2011 IEEE International Conference on Rehabilitation Robotics (ICORR).

[21] Wilson S., Eberle H., Hayashi Y., Madgwick S.O., McGregor A., Jing X., Vaidyanathan R. (2019). Formulation of a new gradient descent MARG orientation algorithm: Case study on robot teleoperation. Mech. Syst. Signal Process..

[22] Valenti R.G., Dryanovski I., Xiao J. (2015). Keeping a good attitude: A quaternion-based orientation filter for IMUs and MARGs. Sensors.

[23] Wu J., Zhou Z., Chen J., Fourati H., Li R. (2016). Fast complementary filter for attitude estimation using low-cost MARG sensors. IEEE Sens. J..

[24] Fan B., Li Q., Liu T. (2018). Improving the accuracy of wearable sensor orientation using a two-step complementary filter with state machine-based adaptive strategy. Meas. Sci. Technol..

[25] Gośliński J., Nowicki M., Skrzypczyński P. (2015). Performance comparison of EKF-based algorithms for orientation estimation on Android platform. IEEE Sens. J..

[26] Li W., Wang J. (2013). Effective adaptive Kalman filter for MEMS-IMU/magnetometers integrated attitude and heading reference systems. J. Navig..

[27] Mazza C., Donati M., McCamley J., Picerno P., Cappozzo A. (2012). An optimized Kalman filter for the estimate of trunk orientation from inertial sensors data during treadmill walking. Gait Posture.

[28] Feng K., Li J., Zhang X., Shen C., Bi Y., Zheng T., Liu J. (2017). A new quaternion-based Kalman filter for real-time attitude estimation using the two-step geometrically-intuitive correction algorithm. Sensors.

[29] Kownacki C. (2011). Optimization approach to adapt Kalman filters for the real-time application of accelerometer and gyroscope signals’ filtering. Digit. Signal Process..

[30] Wu J. (2020). MARG Attitude Estimation Using Gradient-Descent Linear Kalman Filter. IEEE Trans. Autom. Sci. Eng..

[31] Mourcou Q., Fleury A., Franco C., Klopcic F., Vuillerme N. (2015). Performance evaluation of smartphone inertial sensors measurement for range of motion. Sensors.

[32] Cavallo A., Cirillo A., Cirillo P., De Maria G., Falco P., Natale C., Pirozzi S. (2014). Experimental comparison of sensor fusion algorithms for attitude estimation. IFAC Proc. Vol..

[33] Kuti J., Galambos P., Györök G. Adaptive Odometry and IMU Sensor Fusion for KUKA youBot Mobile Robot Using Analytical Time Update. Proceedings of the 2019 IEEE 23rd International Conference on Intelligent Engineering Systems (INES).

[34] Odry Á. An Open-Source Test Environment for Effective Development of MARG-Based Algorithms Supplementary Material. http://appl-dsp.com/faekf/.

[35] Sciavicco L., Siciliano B. (2012). Modelling and Control of Robot Manipulators.

[36] Haidegger T., Kovács L., Precup R.E., Preitl S., Benyó B., Benyó Z. (2011). Cascade control for telerobotic systems serving space medicine. IFAC Proc. Vol..

[37] Haidegger T., Kovács L., Preitl S., Precup R.E., Benyó B., Benyo Z. (2011). Controller design solutions for long distance telesurgical applications. Int. J. Artif. Intell..

[38] Aggarwal P. (2010). MEMS-Based Integrated Navigation.

[39] Papafotis K., Sotiriadis P.P. (2019). MAG.I.C.AL.—A Unified Methodology for Magnetic and Inertial Sensors Calibration and Alignment. IEEE Sens. J..

[40] Sarcevic P., Kincses Z., Pletl S. (2019). Online human movement classification using wrist-worn wireless sensors. J. Ambient. Intell. Humaniz. Comput..

[41] Sarcevic P. Examining the Efficiency of Magnetometers in Movement Classification Systems. Proceedings of the 2020 IEEE 14th International Symposium on Applied Computational Intelligence and Informatics (SACI).

[42] Zmitri M., Fourati H., Prieur C. (2020). Magnetic Field Gradient-Based EKF for Velocity Estimation in Indoor Navigation. Sensors.

[43] Marple L. (1999). Computing the discrete-time “analytic” signal via FFT. IEEE Trans. Signal Process..

[44] Furrer F., Burri M., Achtelik M., Siegwart R. (2016). Robot Operating System (ROS): The Complete Reference (Volume 1).

[45] Meyer J., Sendobry A., Kohlbrecher S., Klingauf U., Von Stryk O. Comprehensive simulation of quadrotor uavs using ros and gazebo. Proceedings of the International conference on simulation, modeling, and programming for autonomous robots.

[46] Kok M., Hol J.D., Schön T.B. (2017). Using inertial sensors for position and orientation estimation. Found. Trends Signal Process..

[47] Gunther M., Dryanovski I. IMU Tools for ROS. wiki.ros.org/imutools.

[48] Lu D.V., Ferguson M., Hoy A., Meeussen W. Robot Pose EKF. wiki.ros.org/robotposeekf.

[49] Madgwick S. (2010). An Efficient Orientation Filter for Inertial and Inertial/Magnetic Sensor Arrays. https://forums.parallax.com/uploads/attachments/41167/106661.pdf.

[50] MathWorks Estimate Orientation through Inertial Sensor Fusion. www.mathworks.com/help/fusion/ug/estimate-orientation-through-inertial-sensor-fusion.html.

[51] Michel T., Genevès P., Fourati H., Layaïda N. (2018). Attitude estimation for indoor navigation and augmented reality with smartphones. Pervasive Mob. Comput..

[52] Jouybari A., Amiri H., Ardalan A.A., Zahraee N.K. (2019). Methods comparison for attitude determination of a lightweight buoy by raw data of IMU. Measurement.

[53] Sarabandi S., Thomas F. (2019). A survey on the computation of quaternions from rotation matrices. J. Mech. Robot..

[54] Odry Á., Kecskés I., Burkus E., Odry P. (2017). Protective Fuzzy Control of a Two-Wheeled Mobile Pendulum Robot: Design and Optimization. WSEAS Trans. Syst. Control.

